# Molecular cloning and expression analyses of porcine MAP1LC3A in the granulosa cells of normal and miniature pig

**DOI:** 10.1186/1477-7827-11-8

**Published:** 2013-02-12

**Authors:** Sang H Kim, Sue Y Hwang, Kwan S Min, Jong T Yoon

**Affiliations:** 1 Institute of Genetic Engineering, Hankyong National University, Ansung, 456-749, Korea; 2 Graduate School of Bio & Information Technology, Hankyong National University, Ansung, 456-749, Korea; 3 Department of Animal Life Science, Hankyong National University, Ansung, 456-749, Korea

**Keywords:** Autophagy, MAP1LC3A, Granulosa cell, Pig, Miniature pig

## Abstract

**Background:**

The members of the microtubule-associated protein 1 light chain (MAP1LC) family, especially those of the LC3 family (MAP1LC3A, B, C), are known to induce autophagy upon localization onto the autophagosomal membrane. In this regard, LC3 can be utilized as a marker for the formation of autophagosomes during the process of autophagy. The aims of this study are to clone porcine MAP1LC3A, and analyze the pattern of its expression in the ovarian tissues of normal and miniature pig ovary in an attempt to understand the distinct mode of apoptosis between two strains.

**Methods:**

Rapid amplification of cDNA ends (RACE) were used to obtain the 5^′^ and 3^′^ ends of the porcine MAP1LC3A full length cDNA. Reverse-transcriptase-PCR (RT-PCR), real-time PCR, and western blot analysis were performed to examine the expression of porcine MAP1LC3A. The localization of MAP1LC3A in the ovary was determined by *In situ* Hybridization and Immunohistochemical staining.

**Results:**

We cloned the full-length cDNA of porcine MAP1LC3A and identified an open reading frame of 980 bp encoding 121 amino acids. Based on its homology to known mammalian proteins (98%) this novel cDNA was designated as porcine MAP1LC3A and registered to the GenBank (Accession No. GU272221). We compared the expression of MAP1LC3A in the Graafian follicles of normal and miniature pigs by *in situ* hybridization at day 15 of the estrus cycle. While normal pigs showed a stronger expression of MAP1LC3A mRNA than miniature pigs in the theca cell area, the expression was lower in the granulosa cells. Immunofluorescence analysis of the MAP1LC3A fusion reporter protein showed the subcellular localization of porcine MAP1LC3A and ATG5 as a punctate pattern in the cytoplasm of porcine granulosa cells under stress conditions. In addition, the expressions of MAP1LC3A and ATG5 were higher in normal pigs than in miniature pigs both in the presence and absence of rapamycin.

**Conclusions:**

The newly cloned porcine MAP1LC3A provides a novel autophagosomal marker in both normal and miniature pig. We demonstrated that the expression of MAP1LC3A in graafian follicle is distinct in normal and miniature pig, which may explain the unique folliculogenesis of miniature pigs.

## Background

Autophagy is a process of programmed cell death (PCD) through which damaged tissues and pathogens are removed. It is also involved in the degradation of abnormal proteins and in the remodeling of cells and tissues. With regard to this, cells are required to carry out an appropriate level of autophagy to maintain their survival as well as for intracellular homeostasis [1-3]. In particular, autophagy is closely associated with the remodeling of cells during the process of tissue formation [4]. A recent report suggested that apoptosis, probably through autophagy, promotes granulosa cells to form atresia in developing follicles [5]. The role of apoptosis during follicular development and atresia formation has been well studied in multi-ovulating mammals such as pigs; however, little is known about the possible involvement of autophagy in these processes [6,7]. In most mammals, autophagy is initiated by the suppression of the IGF-signaling pathway. The process of macroautophagy is also induced by rapamycin through the inhibition of mTOR activity [7]. In the course of autophagy, Beclin-1 first binds to Vps15 followed by the joining of ATG12-ATG5 and LC3 (ATG8), leading to the formation of a double-membrane structure called the autophagic vacuole (AV-1) [8-10]. This initial stage of the autophagosome includes some of the cytosol and the cellular organelles within it. Later, the AV-1 fuses with the lysosomes and develops into a single-membrane second-stage vacuole (AV-II) [11]. The formation of the mature autophagolysosome can be determined by the presence of the surface marker LC3. LC3 is a type of microtubule-associated protein (MAP), which is classified as MAP1LC3A, MAP1LC3B, or MAP1LC3C [12,13]. LC3 is further divided into LC3-I, which remains within the cell, and LC3-II, which binds to the phosphatidylethanolamine (PE) of the initial autophagosomal membrane [12]. LC-II remains in the membrane until the formation of the autophagolysosome [14,15], and thus can be utilized as a marker to determine the activity of autophagy. Mizushima et al. (2001) reported that the expression of LC-II is controlled by ATG5 another potential marker for detecting autophagy [16]. The genes encoding LC3 were cloned in a variety of mammalian species; however, its porcine homolog remains to be identified. In the present study, we determined the full-length nucleotide sequence of porcine MAP1LC3A cDNA by using mixed-base oligonucleotide primers designed based on previously cloned MAP1LC3A from other species. We also compared the expression of MAP1LC3A of granulosa cells in normal and miniature pig.

## Methods

### Collection of porcine ovary tissue

Ovaries were dissected from normal (Landrace, age: 10month, weight: 110±5.1kg) and miniature (Age: 10 month, weight: 26±2.1 kg, Medi Kinetics co., Ltd; Pyeongteak, Korea) pigs were collected from gilts at a slaughterhouse (PyeongNong, Pyeongteak, Korea) and transported in physiological saline supplemented with penicillin G (100 U/ml) and streptomycin sulfate (100 mg/ml) at 37°C, within 2 h to the laboratory. Treated ovaries were either kept in phosphate buffered saline (PBS) (for follicular cell extraction) or stored in liquid nitrogen for molecular analysis. This study was carried out in strict accordance with the recommendations laid out in the Guide for the Care and Use of Laboratory Animals of the National Institutes of Health. The protocol was approved by the Committee on the Ethics of Animal Experiments of the Hankyong National University (Permit Number: 2012–1).

### Granulosa cell culture

Follicles were obtained from freshly isolated ovaries. Fluid from follicles (4–8 mm in diameter) was aspirated using an 18-gauge needle attached to a 5-ml disposable syringe. Follicular aspirates were kept at 37°C and allowed to sediment for 5 min. The sediment was removed, and the remaining was allowed to sediment for a further 30 min, until pellets of aggregated granulosa cells with a uniform size range were created. The supernatant containing mostly single cells was discarded, and the pellets were transferred to non-treated petri dishes. With the aid of a dissecting microscope, all naked oocytes, cumulus oocyte-complexes (COCs), and debris were removed from the aspirate and washed twice in PBS. The sediment was collected into a centrifuge tube and washed several times in PBS, with centrifugation between washes. An aliquot of resuspended cells was manually dissociated using a 1-ml syringe with a 23-gauge needle and counted using a hemocytometer to determine the cell density.

Granulosa cells were plated in DMEM and 10% fetal bovine serum (FBS; Life Technologies, Carlsbad, CA) at a density of 3.5 × 10^7^ in a T-25 tissue culture flask (Becton Dickinson and Co., Franklin Lakes, NJ) for 16 h, to facilitate the attachment of the granulose cells to the bottom of the flask. Once cells were attached, the medium was replaced with fresh DMEM containing 10% FBS and cultured for 24 h or 48 h at 37°C in a humidified atmosphere of 5% CO_2_ and 95% air. Following this, 100 nM of rapamycin solution was added to each well and the cells were incubated for 24 h at 37°C in a humidified atmosphere of 5% CO_2_ and 95% air. After 48 h of culture, samples of both normal untreated cells and rapamycin-treated cells were collected for immunoassay.

### Cloning of MAP1LC3A cDNA

The amino acid sequences of human LC3A (GenBank Accession No. AF276658) were used to search the porcine expressed sequence tag (EST) database at GenBank (http://www.ncbi. nlm. nih.gov). Three homologous EST clones were obtained and manually assembled into a contig. Two pairs of primers (named LC3A) were designed from this contig sequence and used for the cloning of porcine MAP1LCA cDNA by reverse transcription PCR (RT-PCR) of the total RNA from porcine ovary (see Table 1 for primers).

**Table 1:** Primers used for the full-length cloning and expression of the porcine MAP1LC3A gene

**Primer name**	**Sequence**	**Size**
*cDNA cloning primers*		
*GeneRacer 3*^*′*^*-primer*	*5*^′^*GCTGTCAACGATACGCTACGTAACG 3*^′^	
*GeneRacer 3*^**′**^*-nested primer*	*5*^′^*CGCTACGTAACGGCATGACAGTG 3*^′^	
*GeneRacer 5*^*′*^*-primer*	*5*^′^*CGACTGGAGCACGAGGACACTGA 3*^′^	
*GeneRacer 5*^*′*^*-nested primer*	*5*^′^*GGACACTGACATGGACTGAAGGAGTA 3*^′^	
*5*^′^*RACE-Rv primer 1*	*5*^′^*TGTCCAGGACTGGCAGCTGCTTCT 3*^′^	
*5*^′^*RACE-Rv primer 2*	*5*^′^*CCAACTCGCTCATGTTGACATGGT 3*^*′*^	
*3*^′^*RACE-Fw primer*	*5*^′^*ATCGAGCGCTACAAGGGTGAGAAGC 3*^′^	
*Gene expression primers*		
*Porcine GAPDH Fw*	*5*^′^*CCCGTTCGACAGACAGCCGTG 3*^′^	*238*
*Porcine GAPDH Rv*	*5*^′^*CCGCCTTGACTGTGCCGTGG 3*^′^	
*Porcine MAP1LC3A Fw*	*5*^′^*AGAAGCAGCTGCCAGTCCTGGACA 3*^′^	*687*
*Porcine MAP1LC3A Fw*	*5*^′^*CAGGCAGGCCTGAGCAATCTTTATT 3*^*′*^	
*Porcine ATG5 Fw*	*5*^′^*AGAGAAGTCTGTCCTTCCGCAGTCG 3*^′^	*241*
*Porcine ATG5 Rv*	*5*^′^*AAGCAGAAGGGTGACATGCTCTGGT 3*^′^	

The PCR amplification conditions were as follows: 100 ng/μl of reverse-transcribed cDNA was amplified in a 25 ul volume containing 2.5 ul of 10× PCR buffer, 1 ul of 2.5 mM dNTPs, 2 U of *Taq* polymerase, and 1ul of the 10nM each specific primers. PCR amplifications were performed in a PTC-200 DNA Engine thermocycler (MJ Research, Waltham, MA) for 40 cycles of denaturation at 95°C for 35 s, annealing at 60°C for 35 s, extension at 72°C for 40 s, followed by a final extension step at 72°C for 10 min. Amplified PCR products were TA-cloned into a pGEM-T vector and sequenced using the BigDye terminator sequencing kit and the ABI377 sequencer (PerkinElmer Life Sciences, Boston, MA) according to the manufacturer’s instructions.

### 5^′^ and 3^′^ RACE

5^*′*^ RACE was performed using a GeneRacer kit (Invitrogen). Total RNA was treated with calf intestinal phosphatase (CIP) to remove the 5^*′*^ phosphates to eliminate truncated mRNA from subsequent steps involving ligation with the GeneRacer RNA Oligo. Dephosphorylated RNA was then treated with tobacco acid pyrophosphatase (TAP) to remove the 5^′^ cap structure from the intact full-length mRNA. This treatment leaves a 5^′^ phosphate, which is required for the ligation of the GeneRacer RNA Oligo to the 5^′^ end of the mRNA by using T4 RNA ligase. The 5^′^ RACE primers were designed according to the nucleotide sequence of a previous PCR product. A population of the mRNAs was transcribed into cDNA with an adaptor-primer containing a poly (dT) tract at the 3^′^ end, and an arbitrary sequence of 30–40 nucleotides at the 5^′^ end. Followed by 2 successive 5^′^-RACE PCRs, elongated mRNA was reverse-transcribed into cDNA. 3^′^ RACE was performed using a GeneRacer Kit according to the manufacturer’s instructions.

### *In situ* hybridization of MAP1LC3A mRNA

Digoxigenin-labeled antisense and sense complementary MAP1LC3A RNA probes were prepared as previously described. Using recommended protocols, *in situ* hybridization was performed using a digoxigenin-labeled hybridization kit (Roshe, Mannheim, GER). For hybridization, ovarian tissues were sliced into 10-μm sections. Digoxigenin-labeled probes (200 ng/ml) were hybridized to the ovarian tissue sections by using RiboHybe hybridization solution (TOYOBO, Osaka, JPN) at 65°C for 16 h. Sections were washed in 2× SSC for 5 min at 37°C and were fixed with 60% formamide in 0.2× SSC, which was applied 3 times for 5 min at 37°C. After fixation, the sections were washed in 2× SSC for 5 min at 37°C. The probes were detected with an anti-digoxigenin antibody (1:200) in blocking solution and NBT/BCIP stock solution (0.18 mg/ml BCIP, 0.34 mg/ml NBT, and 240 μg/ml levamisole). The samples were incubated for 16 h at room temperature. The slides were briefly dipped in fresh xylene, and a drop of permount (Fisher, PA) was then applied to the samples, which were finally covered with coverslips.

### Real-time PCR of MAP1LC3A and ATG5

Total RNA was extracted from granulosa cell scrapings by using TRIzol reagent (Invitrogen, CA). The extracted product was treated with DNAse (Ambion, Austin, TX) as per the manufacturer’s instructions and then quantified by UV spectrophotometry. First-strand cDNA synthesis was achieved by reverse transcription of mRNA by using an Oligo (dT) primer and SuperScript^tm^ II Reverse Transcriptase (Invitrogen, Grand Island, NY). Real-time PCR using Line-gene K (Bioer Technology, Tokyo, JPN) was then performed in a final reaction volume of 25 μl with SYBR Green (TOYOBO, Osaka, JPN). The primers used for PCR are shown in Table 1. The PCR conditions were as follows: 10 min at 94°C, followed by 39 cycles of 30 s at 94°C, 30 s at 60°C or 65°C, 55 s at 72°C, and a final extension step of 5 min at 72°C. Rotor-Gene Real-Time Software 6.0 was used to analyze the results by using a cycle threshold (Ct) to assess the semi-log amplification plot. Finally, the relative gene expression was analyzed using the 2-ΔΔCt method normalized to porcine glyceraldehyde-3-phosphate dehydrogenase (GAPDH) mRNA levels [17].

### Western blot

Approximately 30 μg of protein was electrophoretically separated in duplicate on a 13% gel by sodium dodecyl sulfate-polyacrylamide gel electrophoresis (SDS-PAGE), and transferred onto an immunoblot PVDF membrane (Bio-Rad). The membrane was incubated in blocking buffer (5% non-fat dry milk) overnight at 4°C and washed 3 times every 10 min with washing buffer (0.1% v/v Tween 20, 50 mM Tris–HCl pH 7.6, and 200 mM NaCl). The membrane was then incubated for 2 h with anti-rabbit MAP1LC3A monoclonal antibody (diluted 1:1000; Abcam, MA), anti-rabbit ATG5 polyclonal antibody (diluted 1:1000; Abfrontier, Seoul, Kor). Following this, the membranes were washed 3 times for 15 min, with 1× TBS-T buffer, and then incubated for 2 h with HRP-conjugated anti-rabbit and anti-mouse secondary antibodies (diluted 1:5000). The membrane was then developed in a dark room following exposure for 5 min with the detection reagent from an ECL-detection kit. The detection reagent was drained off and the membrane was exposed with a sheet of diagnostic film in the film cassette for 1–30 min.

### Detection of the MAP1LC3A and ATG5 by immunofluorescence

Immunodetection of MAP1LC3A and ATG5 was performed on granulosa cells mounted on silanized slides. Granulosa cells were cultured on sterilized glass coverslips, fixed with 4% paraformaldehyde, and blocked with 0.1% BSA in PBS. Slides were then incubated with monoclonal antibodies that specifically recognize the active forms of MAP1LC3A and ATG5 at a 1:150 dilution. After washing, the slides were incubated with an anti-rabbit IgG conjugated to Alexa 488 or Alexa 594 (Molecular Probes, Eugene, OR).

Nuclei were counter-stained with 1ug/ml Hoechst 33258, and slides were treated with fluorescent mounting medium (Dako, Carpinteria, CA). Images were acquired using an Olympus AX70 fluorescence microscope fitted with a CCD color camera.

### Statistical analysis

Data were analyzed using the *t*-test with the Statistical Analysis System software (SAS Institute, version 9.4, Cary, NC). Differences among the treatment means were determined using Duncan’s multiple range tests.

## Results

### Cloning of the full-length porcine MAP1LC3A gene

**Figure 1:**
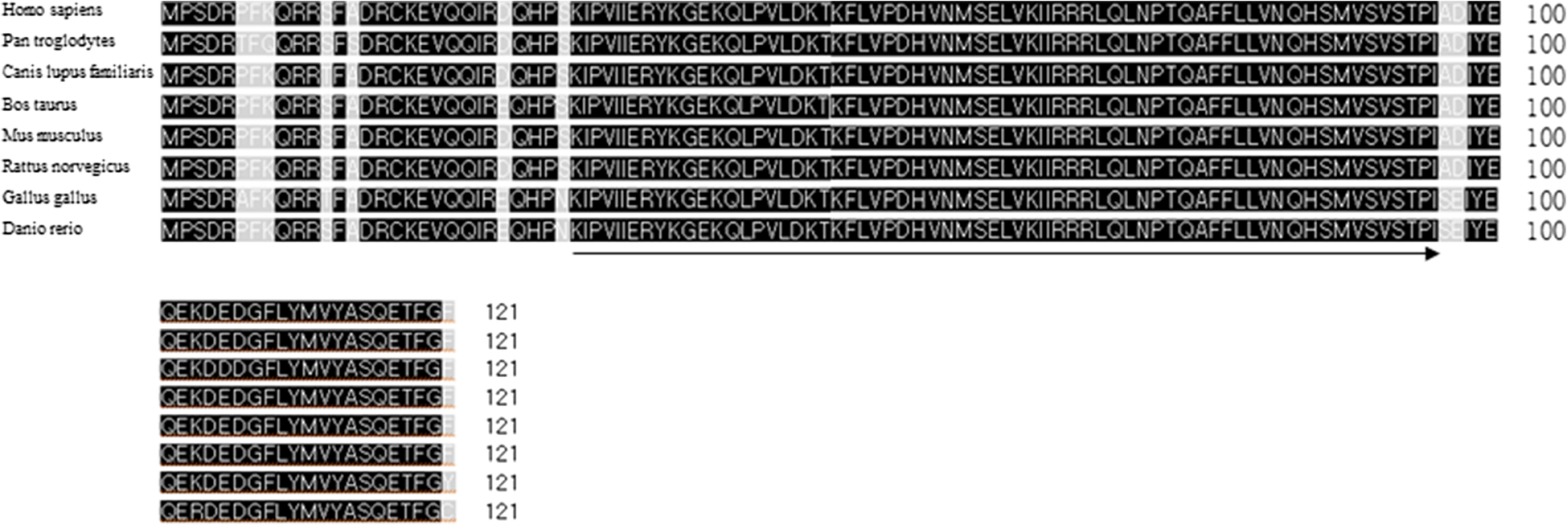
**Amino acid sequence comparison of mammalian MAP1LC3 proteins.** Proteins used for alignments were from *Homo sapiens* (NP_115903.1)*; Pan troglodytes* (XP_001159668.1)*; Canis lupus familiaris* (XP_534391.2)*; Bos taurus* (NP_001039640.1)*; Mus musculus* (NP_080011.1)*; Rattus norvegicus* (NP_955794.1)*; Gallus gallus* (XP_417327.2)*;* and *Danio rerio* (NP_999904.1). Identical residues are shaded in black, and similar residues are shaded in gray. Arrows indicate the primers used for cloning pig homologs.

**Figure 2:**
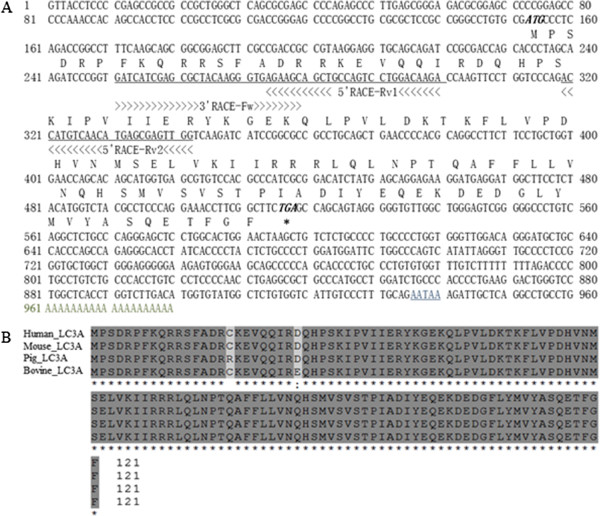
**Full-length cDNA and ORF of porcine MAP1LC3A. A.** Full-length cDNA and ORF of porcine MAP1LC3A. **B.** Comparison of the deduced amino acid sequences of the MAP1LC3A ORF with known mammalian proteins.

To design the primers for cloning the porcine *MAP1LC3A* gene, we compared the nucleotide sequence of homologous genes that have been identified in other vertebrates, such as humans, dog, cow, mouse, rat, chicken, and the zebra fish, in a multiplex alignment (Figure 1). PCR primers were designed to encompass the most conserved region between Lys (K)-30 and Ile (I)-95. PCR amplification using these primers produced a 710-bp fragment of porcine cDNA. The rest of the gene was obtained by 5^′^ and 3^′^-RACE, which were combined together to generate a 980-bp full-length sequence with an open reading frame of 121 amino acids. We found that the deduced amino acid sequence showed over 98% homology to the human, mouse, and cow proteins. In the regions upstream of Gly-120 at the C-terminus, porcine MAP1LC3A was 100% identical to those of other mammals with an exception of some residue variation at Cys-16, which was replaced by Arg in humans, mouse, and cow. Such a high degree of structural homology strongly suggested that this newly cloned protein would perform the same function as the known mammalian MAP1LC3As, and was thus named as porcine MAP1LC3A (Figure 2). The sequence information of both cDNA and amino acids has been deposited in the GenBank (No. GU272221).

### Expression of MAP1LC3A in porcine follicles

**Figure 3:**
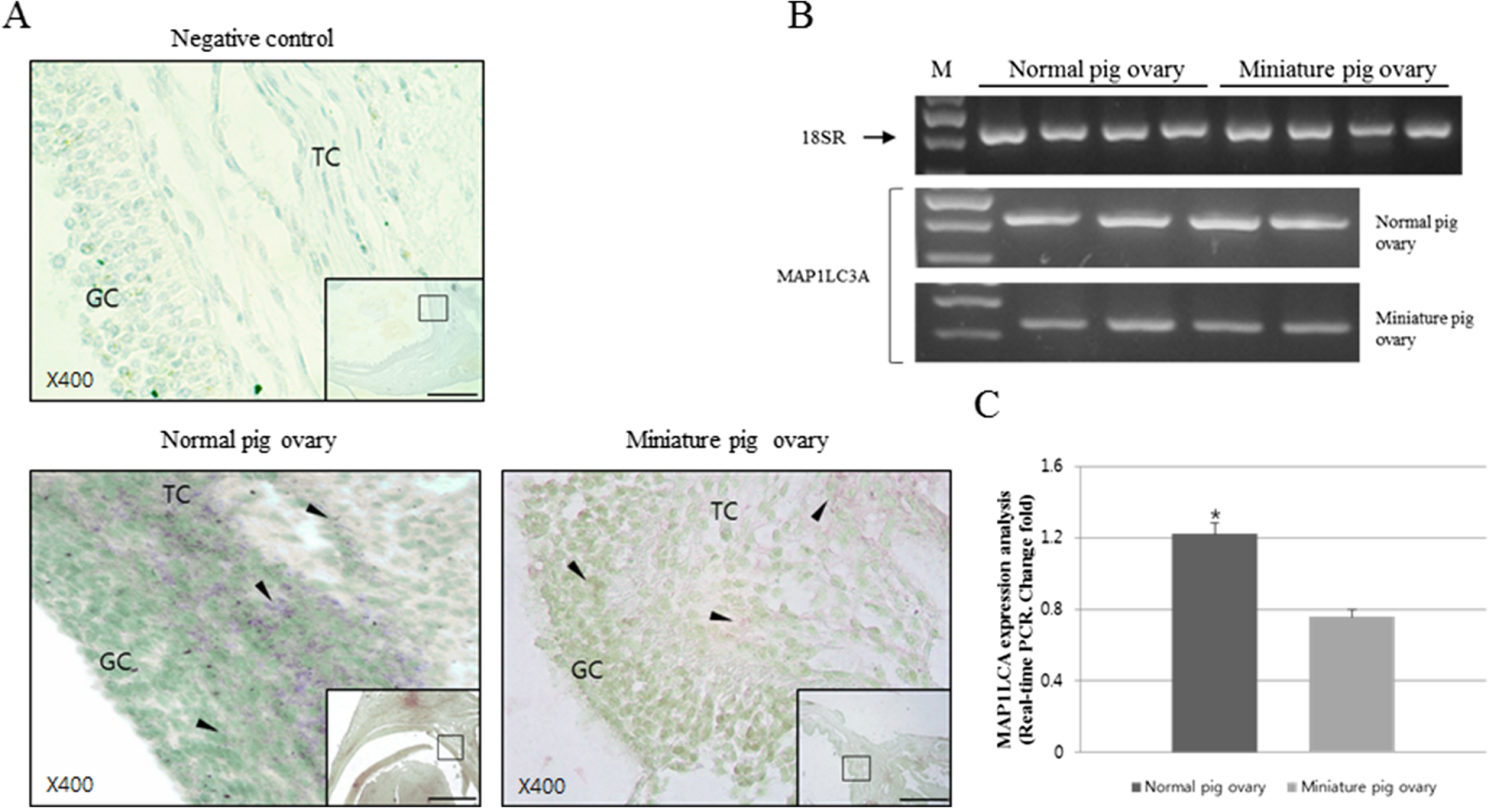
**In situ detection and expression analyses of MAP1LC3A mRNA in the ovary. A.***In situ* hybridization of MAP1LC3A mRNA in the Graafian follicle of normal and miniature pigs. Arrows indicate positive cells. GC: granulosa cells; TC: theca cells. Prehybridization solution was used as the control for the Graafian follicle below the negative panel. **B.** Reverse transcription PCR and **C.** Real-time PCR of total RNAs from normal and miniature pig ovaries. Experiments were repeated 3 times, and data are expressed as mean ± standard error (p < 0.05). Black bar=100 um in all figures.

The expression of MAP1LC3A mRNA was examined by *in situ* hybridization in the Graafian follicles of pigs and miniature pigs at day 15 of the estrus cycle. Normal pigs exhibited higher levels of MAP1LC3A mRNA in the Graafian follicles than miniature pigs (Figure 3). The site of expression was also different between the 2 species. Although MAP1LC3A mRNA was more strongly expressed in the theca cell area of normal pigs than in that of miniature pigs, the expression was lower in the granulosa cells (Figure 3A). A higher expression of MAP1LC3A was also confirmed through conventional RT-PCR as well as through real-time PCR analyses of 4 animals from each group (Figure 3B). Therefore, MAP1LC3A was more abundant in the theca interna layer, which undergoes substantial remodeling through the processes of follicular development and expansion, than it was in the granulosa cell wall.

### Expression pattern of autophagy marker proteins in follicular cells

**Figure 4:**
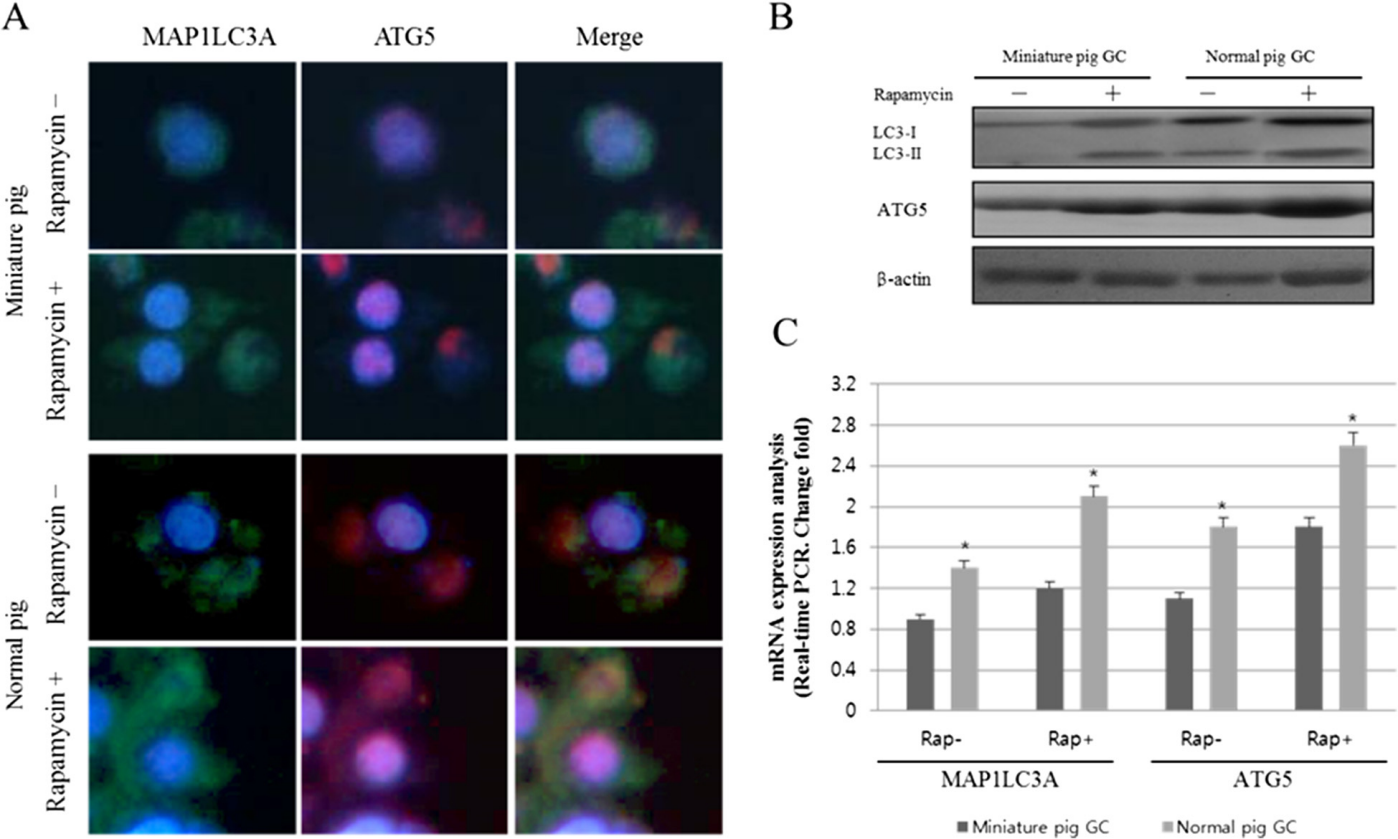
**Effect of rapamycin treatment on the expression of MAP1LC3A and ATG5 protein expression in cultured granulosa cells. A.** Immunofluorescence staining of cultured granulosa cells. **B.** Western blot analysis of the porcine MAP1LC3A protein. **C.** Real-time PCR of porcine MAP1LC3A mRNA. Data represent the mean ± standard error of 3 individual experiments (p < 0.05).

Next, we examined the effect of rapamycin treatment on the expression of the autophagy marker proteins, MAP1LC3A and ATG5, in the follicular cells of pigs and miniature pigs. Granulosa cells exhibited an intrinsically higher expression of both markers, but this expression was even more enhanced upon rapamycin treatment (Figure 4). When the expression levels were compared between pigs and miniature pigs, marker expression was higher in normal pigs in the presence and in the absence of rapamycin (Figure 4A). We also assessed the changes in the expression of LC3 subtypes by western blot analysis (Figure 4B). In miniature pig granulosa cells, rapamycin elevated both LC3A-I and LC3A-II levels compared to those in the non-treated controls. Treating the granulosa cells of normal pigs with rapamycin resulted in an increase in LC3A-I but it actually lowered the expression of LC3A-II, probably due to the inherently robust expression of LC3A-II in pigs. Granulosa cells expression of ATG5 in normal pigs was higher than that in miniature pig cells, which was highly induced by rapamycin treatment. The changes and differences in the expression of these marker proteins were well reflected in the analyses of the corresponding mRNAs (Figure 4C).

## Discussion

Autophagy is known to affect the process of development by enabling the remodeling of tissues and cells. A certain level of autophagy is essential for the maintenance of cellular survival and homeostasis [2,3]. The importance of appropriately initiated autophagy is particularly emphasized in the process of mammalian follicular development. The close association of apoptosis with folliculogenesis has been extensively studied [6,18]; however, a great paucity in the knowledge concerning the role of autophagy during porcine follicular development remains. It is well known that miniature pigs are distinct from normal pigs in many aspects of follicular development, as well as in the mode of ovulation and luteinization. Many of these distinctions between the 2 species are believed to arise from genetic differences in the factors governing the process of female reproduction [19,20].

During oocyte development, the ovarian follicles undergo repeated remodeling, and they eventually regress into the corpus luteum where they disintegrate through apoptosis [21,22]. According to a recent report, autophagy appears to serve as a major mechanism for the disintegration of dying follicles [5]. To this end, the distinct pattern of MAP1LC3A that we observed in miniature pig follicles may provide a clue to understanding the unique pathway of folliculogenesis in this animal. For example, we observed that MAP1LC3A was more abundant in the theca interna layer than it was in the granulosa cell wall. This result is reminiscent of a previous report by Duerrschmidt et al. (2006) which suggested that autophagy, as well as apoptosis, is involved in follicular development [18]. One intriguing fact in this report was that the expression pattern of autophagy markers in the Graafian follicles was different between pig and miniature pigs. Similar differences were also observed among cells cultured *in vitro*. Furthermore, treating the cells with rapamycin, a chemical inducer of autophagy, had a different effect on the expression of autophagy markers in the pig and miniature pig cells. Together, these results suggest that miniature pigs may employ a unique control of autophagy during follicular development, thereby indicating that pigs and miniature pigs are different in terms of their modes of reproduction [23-27]. A more detailed molecular analysis of the cellular signaling pathways controlling the process of autophagy will provide valuable information for improving the reproduction rates of miniature pig oocytes.

## Conclusions

We report the first cloning of a full-length porcine MAP1LC3A gene. The open reading frame of porcine MAP1LC3A cDNA consists of 980 bp (encoding 121 amino acids). Based on its homology to known mammalian proteins (98%) this novel cDNA was designated as porcine MAP1LC3A and registered to the GenBank (Accession No. GU272221). In addition, the expressions of MAP1LC3A and ATG5 were higher in normal pigs than in miniature pigs in the presence and in the absence of rapamycin. These results indicate that MAP1LC3A can be used as an autophagosomal marker in porcine follicular cells. Based on the expression of this novel marker protein, we propose that autophagy plays a role in the maintenance of follicular development at least partially by regulating the remodeling system in porcine follicular cells.

## Competing interests

The authors declare that they have no competing interests.

## Authors’ contributions

SHK performed the experiments. SHK and SYH performed IHC. SHK and SYH drafted the manuscript. SHK, SYH, KSM, and JTY designed the study, supervised the experimental work, and revised the manuscript. These authors contributed equally to this paper. All authors read and approved the final manuscript.
